# Effect of Ultra-High-Pressure Homogenization Processing on the Microbiological, Physicochemical, and Sensory Characteristics of Fish Broth

**DOI:** 10.3390/foods11243969

**Published:** 2022-12-08

**Authors:** Sonia Genuina Moisés, Buenaventura Guamis, Artur Xavier Roig-Sagués, Idoia Codina-Torrella, Maria Manuela Hernández-Herrero

**Affiliations:** 1 Departament de Ciència Animal i dels Aliments, Facultat de Veterinària, Centre d’Innovació, Recerca i Transfèrencia en Tecnologia dels Aliments (CIRTTA), XaRTA, TECNIO-CERTA, MALTA-Consolider Team, Universitat Autònoma de Barcelona, 08193 Bellaterra, Spain; 2 Departament d’Enginyeria Agroalimentària i Biotecnologia, Edifici D4C, Esteve Terradas, 8, 08860 Castelldefels, Spain

**Keywords:** ultra-high-pressure homogenization, UHPH, fish broth, microbial stabilization, physical stability

## Abstract

The effect of ultra-high-pressure homogenization (UHPH) treatments at 300 MPa at inlet temperatures (T_i_) between 45 and 75 °C on the microbiological, physical, and sensorial characteristics of fish broth was evaluated. Before the application of UHPH treatments, different fish broth formulations were tested, selecting the formula with the best organoleptic and nutritional characteristics and the lowest cost, containing 45% monkfish heads and rock fish in the same proportion. The microbiological shelf-life of fish broth during cold storage at 4 and 8 °C was extended by a minimum of 20 days by applying UHPH treatments at inlet temperatures (T_i_) between 45 and 65 °C. Fish broth UHPH-treated at T_i_ = 75 °C was microbiologically sterile during storage at 4 °C, 8 °C, and room temperature. Fish broth UHPH-treated was physically stable, significantly reducing the particle size. Color showed higher luminosity and lower yellowness as the inlet temperature increased. In fish broth UHPH-treated at T_i_ = 75 °C, selected for its microbiological stability, no differences were observed in the nutritional composition, antioxidant activity, and sensorial perception compared to untreated fish broth. Hence, UHPH treatments showed to be an alternative to preserving fish broth with an improved microbiological shelf-life and good sensorial characteristics.

## 1. Introduction

In recent times, essential approaches have been implemented to reduce waste and recover usable materials from fish during processing. In the fishery product industry, taking advantage of those species or parts of fish with less commercial value and by-products, including the head, guts, bones, skin, fins, frame, and meat adhered to the bones and skin, can be used to elaborate other food products [1].

Several factors must be considered when developing and marketing these products, such as processing conditions, changing consumer demands, cultural and nutritional needs, and technological innovation [2]. In addition, other factors to consider are the quality of food, such as taste, appearance, texture, and smell; socioeconomic factors, such as availability, price, and culture; biological factors, such as energy and nutrient requirements; and psychological factors, including behaviors, moods, and attitudes toward food that influence food choices among consumers [1].

Nowadays, there is a growing demand for ready-to-eat (RTE) or ready-to-cook meals. The growing popularity of ready-to-eat foods over traditional foods is due to the reduced time spent in the kitchen and changes in family structures. Fish broth is a popular food worldwide and suitable for all ages. However, preparing fish broth is time-consuming and troublesome, so it is advantageous to serve it as a ready-to-eat food [3]. It may be consumed as a low-caloric and light RTE meal. However, it is an excellent source of vital nutrients for human nutrition, with high levels of macro- and micronutrients. It also provides phospholipids, polyunsaturated fatty acids, and various essential minerals that play a valuable role in a healthy and balanced human diet [4]. Nevertheless, fish broth is highly perishable due to a pH close to neutral and high-water activity, requiring the application of preservation technologies to improve safety and extend the limited shelf-life of RTE foods to maintain the optimal characteristics for consumption [1]. One of the control strategies or measures is to apply the emerging ultra-high-pressure homogenization (UHPH) technology. This technology allows microbial inactivation and improves the colloidal stability of fluid foodstuffs, maintaining, in most cases, both nutritional and sensory characteristics of untreated products. It is based on the same principle as conventional homogenization but can work at pressures up to 400 MPa. The physical phenomena (e.g., cavitation, pressure drop, and shear stress) that fluid suffers from when passing through the high-pressure valve gap, in combination with the sudden temperature jump, promote significant changes in the product characteristics, such as the disruption of vegetative microorganisms [5] and, in some cases, the reduction in spore counts [6,7,8]. In addition, different studies have pointed out that UHPH improves the colloidal stabilization of these liquid foods through particle size reduction and induced changes in the functional properties [9,10,11].

UHPH technology has already been evaluated, with good results in the microbiological, nutritional, chemical, and physical properties of different liquid food matrices, such as eggs, milk, vegetable beverages based on soy, almonds, tiger nuts, and apple, orange, and grape juices [9,12,13,14,15,16,17,18]. Nevertheless, results obtained in these studies have demonstrated that the effect of this technology depends, to some extent, on the characteristics of the food matrix. For this reason, it is interesting for the food industry to investigate the application of UHPH in other liquid foods.

Considering that the application of UHPH in fish broth has not been studied yet, the purpose of this work was to evaluate the effect of the application of UHPH treatments at 300 MPa at inlet temperatures between 45 and 75 °C on the microbiological, physical, and sensorial characteristics of fish broth. Previously, to minimize the variability of the results related to the ingredients used, especially from fish products, and of the elaboration process, a standardized fish broth formulation from by-products or discards from the fishing industry was developed. The main factors for its selection were organoleptic and nutritional characteristics and the lowest cost.

## 2. Materials and Methods

The experimental flowchart for explaining the section of the best fish broth formulation and for evaluating the effect of the application of ultra-high-pressure homogenization (UHPH) treatments at 300 MPa at inlet temperatures (T_i_) between 45 and 75 °C on the microbiological, physical, and sensorial characteristics of fish broth is shown in Figures S1 and S2.

### 2.1. Raw Material Purchase and Fish Broth Making

The fishing products for preparing fish broth were purchased from a local fish industry located in El Prat de Llobregat (Barcelone, Spain). The products were supplied frozen in 6 kg boxes and stored at −18 °C until use. The products supplied were monkfish heads (*Lophius* spp.) caught in the Northeast Atlantic FAO area; pieces of hake (*Merluccius* spp.) caught in the Mediterranean Sea; and rock fish for soup consisting of pelagic fish of different sizes, also caught in the Mediterranean Sea, such as fish of the *Triglidae* family (*Chelidonichthys* spp., *Trigloporus lastoviza*, or *Trigla lyra*), spotted weever (*Trachinus* spp.), conger eel (*Conger conger*), spotted flounder (*Citharus linguatula*), the *Scorpaena* genus, red mullet (*Mullus* spp.), crab (*Pachygrapsus marmoratus*, *Panapeus africanus* and *Xantho* spp.), and Norway lobsters (*Nephrops norvegicus*). Seasoning spices and vegetables, such as salt, sunflower oil, peppercorns, dried oregano, onion, carrot, leek, celery, and parsley, were purchased from a retail chain.

In total, 13 formulations with different compositions of fish, seasoning spices, and vegetables were evaluated (Table 1). A base broth was prepared according to the recipe described by *Corpus del Patrimoni Culinari Català* [19]. First, decalcified water, sunflower oil, salt, and lemon were boiled, and after 5 min, unfrozen fish and vegetables (washed, peeled, and chopped), such as onion, carrot, leek, and celery, were added. When the boiling temperature was reached (~100 °C), broth was cooked for 30 min, continually eliminating all the foam formed. In the last 5 min of cooking, parsley, oregano, and peppercorns were added. At the end of the process, the largest components were removed with a spatula and the smallest ones were separated by filtration using a stainless-steel vibrating sieve with a mesh size of 100 μm (Figure S1).

### 2.2. UHPH Treatments

UHPH treatments were performed using an ultra-high-pressure homogenizer at a flow rate of 15 L/h (model: DRG No. FPG11300:400 Hygienic Homogenizer, Stansted Fluid Power Ltd., Harlow, UK) at 300 MPa and an inlet temperature (T_i_) of 45, 55, 65, and 75 °C. The high-pressure homogenizer system consisted of two intensifiers driven by a hydraulic pump, a high-pressure homogenization valve, and two spiral-type heat exchangers located before the machine entrance and after the high-pressure valve (Garvía, Barcelona, Spain). The inlet temperature (T_i_), the temperatures before and after the UHPH valve (T_1_ and T_2_, respectively), and the outlet temperature (T_3_) were monitored during the treatments. In this study, T_2_ corresponded to 110.00 ± 1.00, 114.00 ± 1.15, 118.67 ± 0.58, and 128.00 ± 1.00 for the 300 MPa treatments at 45, 55, 65, and 75 °C inlet temperatures, respectively, in which the product remained for <0.5 s [20]. The outlet temperature (T_3_) in all experiments was approximately 25 °C. All samples were collected in sterile glass bottles of 1 L capacity with twist-off caps (Apiglass Envases y Material Apícola, S.L., Barcelona, Spain) inside a laminar-flow cabin (Mini-V cabin, Telstar Technologies, SL, Terrassa, Spain). For the microbiological study, samples were stored at 4 and 8 °C and room temperature (20–25 °C) for 20 days, and analyses were carried out on days 1, 3, 5, 10, 15, and 20. Sensorial analyses were performed on day 1. For the rest of the analyses, after the treatments, samples were stored at 4 or −80 °C until being analyzed.

### 2.3. Microbiological Analyses

Mesophilic aerobic and sporulated microorganisms were enumerated in Plate Count Agar (PCA; Oxoid, Basingstoke-Hampshire, UK) after incubation at 30 °C for 48–72 h. For counting mesophilic aerobic spores, the sample was previously treated at 80 °C for 10 min and then quickly cooled in ice water. *Enterobacteriaceae* were plated in Violet Red Bile Glucose (VRBG; Oxoid) agar and incubated for 24 h at 37 °C. The quantification limit of all counts was 1 cfu/mL. In addition, sterility tests were performed, incubating the fish broth sample (100 mL) at 30 and 55 °C for 21 days. During this period, visual recognition was performed to assess whether phase separation, gelation, or coagulation occurred. In addition, at the end of the time, microbiological analyses were performed on Plate Count Agar (PCA; Oxoid), incubating at 30 and 55 °C for 48–72 h.

### 2.4. Sensorial Analyses

Depending on the sensory analysis method applied, 12 to 18 trained judges were selected from among the Department of Animal and Food Science staff of the *Universitat Autònoma de Barcelona* (UAB) based on their experience in the organoleptic evaluation of food products and their habitual consumption of fish broth. First, we determined the sensory characteristics of the base broth (fish broth 1) using the UNE 87017:1992 method [20]. Four parameters related to the perception and definition of olfactory-gustatory qualities were applied: the degree of intensity of the components present, the aftertaste, the persistence, and the overall impression (description of the degree of harmony as the sample was perceived). The first three parameters were evaluated on an intensity scale from 0 to 5, and the fourth parameter was evaluated on a 3-point scale. The sensorial analysis based on the UNE-ISO 8587:2010 method [21] was applied to optimize the content of vegetables and seasoning spices and the kind and quantity of fish species used. The ordering of the samples was based on the overall impression, considering the description of the degree of harmony perceived in appearance-flavor-consistency on a 3-point scale. Finally, a triangular test based on the UNE-EN ISO 4120:2008 method [22] was applied to evaluate perceptible sensory differences between untreated and fish broth UHPH-treated at T_i_ = 75 °C (UHPH-75), in which sterility was achieved. The triads were presented randomly in three different sessions to the same jury members, performing 30 evaluations.

### 2.5. Physicochemical Composition of Fish Broths

The analytical methods used were those proposed by the Association of Official Agricultural Chemists (AOAC). Total solids were determined using gravimetric determination by drying the samples in an oven at 105 °C (Method 952.08), ashes in a muffle at 550 °C (Method 938.08), and protein content by initial determination of total nitrogen in the raw material using the Kjeldahl method, followed by multiplication by a conversion factor of 6.25 [23]. The pH was determined using a potentiometric method (pHmeter Crison micropH 2001, Alella, Spain). For lipid extraction and purification, the accelerated solvent extraction system was used with ASE 200 equipment (Dionex and Fisher Scientific, Sunnyvale, CA, USA). For this, 2 g of fish broth and washed sea sand were added in a 2:1 ratio to 11 mL stainless-steel extraction cells lined with a cellulose filter. After evaporating the solvents (petroleum ether and isopropanol in a 3:2 ratio) using nitrogen injection, they were placed in a heat block with a temperature gradient of 1 °C per minute from 80 to 105 °C, for 35–40 min. The percentage of lipids was calculated using the difference in weight.

### 2.6. Determination of Total Phenols and Antioxidant Capacity

The procedure used to prepare the extract was based on the method described by Ozgen et al. (2006) [24]. Initially, the samples were centrifuged at 12,000× *g* for 30 min at 4 °C (Sigma 4K15, Sigma, Osterode am Harz, Germany). The supernatant was diluted in distilled water in a ratio of 1:5 to analyze the content of total phenols and antioxidant capacity using ferric-reducing antioxidant power assay (FRAP) and in a ratio of 1:10 for antioxidant activity—Trolox-equivalent antioxidant capacity (TEAC). Total phenols were determined according to the method described by Velioglu et al. (1998) [25] using the Folin–Ciocalteu reagent, reading at an absorbance of 725 nm (Cecil Instruments, Cambridge, UK) using gallic acid (Sigma-Aldrich, St. Louis, MO, USA) as a standard. The calibration curve was established in the range of 10–250 mg/L; the results were expressed in milligram equivalent of gallic acid (mg EAG) per liter of fish broth.

Two spectrophotometric methods were used to evaluate the antioxidant capacity: the Trolox-equivalent antioxidant capacity (TEAC), determined according to the method described by van den Berg et al. (2000) [26], reading absorbance at 734 nm using a spectrophotometer (Cecil Instruments, Cambridge, UK). The ferric-reducing antioxidant capacity (FRAP) was determined according to the method described by Benzie and Strain (1996) [27] and modified according to the method described by Velázquez-Estrada (2013) [28]. Absorbance was measured at 595 nm (ATTC 340, S.L.T. Labinstruments, Salzburg, Austria) using a calibration curve between 25 and 250 μM with Trolox, which corresponds to the chemical compound 6-hydroxy-2,5,7,8-tetramethyl-chroman-2-carboxylic acid (Sigma-Aldrich), expressing the results in mM of Trolox equivalent (TE) for both analytical methods.

### 2.7. Determination of Particle Size

For particle size determination, Beckman Coulter equipment (LS 13 320 series, Beckman Coulter, Fullerton, CA, USA) was used to generate laser diffraction capable of establishing the distribution and size of the particles in the broth formulations. The refractive index used for the dispersed phase was 1.360, and the correspondent to water was 1.334. The particle size distribution was characterized according to the parameters of the surface moment mean diameter or Sauter mean diameter (d_3.2_; particle diameter that has the same specific surface as that of the full distribution) and the volume or mass moment mean diameter or the De Brouckere mean diameter (d_4,3_; diameter of the sphere of equivalent volume to measured particles). D_50_ and D_90_ corresponded to 50% and 90% of particles under the reported particle size.

### 2.8. Color

Color was determined using a Hunter Lab colorimeter (MiniScan XETM, Hunter Associates Laboratory Inc., Reston, VA, USA). Color coordinates were measured using 50 mL of each fish broth sample warmed at 20 °C, with an illuminant of D65 and a standard observer angle of 10°. The colorimeter was calibrated with standard black-and-white tiles. Data were acquired in the CIELab color space: L* (luminosity), a* (red-green), and b* (blue-yellow). The total color difference (∆E*) between untreated and UHPH-treated fish broths was calculated by applying the formula:

$$∆E*={\left[∆{L*}^{2}+∆{a*}^{2}+∆{b*}^{2}\right]}^{1/2}$$

### 2.9. Statistical Analyses

Three independent productions were carried out, and samples were analyzed in triplicate. Results were statistically analyzed using one-way variance analysis (ANOVA), using the statistical program IBM SPSS Statistics v23 (IBM Corp., Armonk, NY, USA) to determine the differences between the values obtained. Tukey’s test was performed, considering that the differences were significant at *p* < 0.05. For the interpretation of the sensory tests, the guidelines of the different methods of the UNE-ISO standards were followed. In this paper, the data exposed in the tables and text correspond to the mean ± standard deviation of every determination.

## 3. Results and Discussion

### 3.1. Sensory Development of Fish Broth

Flavor is one of the most critical characteristics of soups and broths. Most flavoring components are soluble in water, such as nucleotides, amino acids, and peptides, which are the main components that contribute to the flavor of fish [29,30]. In the sensory characteristics of the base broth (broth 1), salt, oil, and peppercorns, followed by monkfish, were perceived with greater intensity. Lemon and leek were moderately perceived, while other vegetables and seasoning spices were detected weakly. The overall impression was valued as moderate (value equal to 2); see Table S1 [20]. For optimization of the quantity of vegetables and seasoning spices (Table S2), as well as the type of fish species used, six new formulations of fish broth were prepared with a fixed quantity of monkfish heads (750 g; broths 2 to 7); see Table S3. After that, four more formulations (broths 8 to 11) were prepared with 750 g of monkfish heads, monkfish heads and hake pieces in a ratio of 1:1, monkfish heads and rock fish in a 1:1 ratio, and monkfish heads, hake pieces, and rock fish in a 1:1:1 ratio (Table S3). Finally, to reduce the cost of fish broth, the quantity of the selected fish broth with rock fish and monkfish heads (broth 10) was reduced to 450 and 600 g (broths 13 and 14, respectively); see Table S4. After statistical analyses according to the UNE-ISO 8527:2010 method [21], fish broth 13 containing 156 g of vegetables, 1.8 g of seasoning spices, and 450 g of monkfish heads and rock fish in a 1:1 ratio was selected, considering the intensity of the taste and smell attributes and the overall impression parameters.

### 3.2. Effect of UHPH on Microbiological Quality

**Figure 1:**
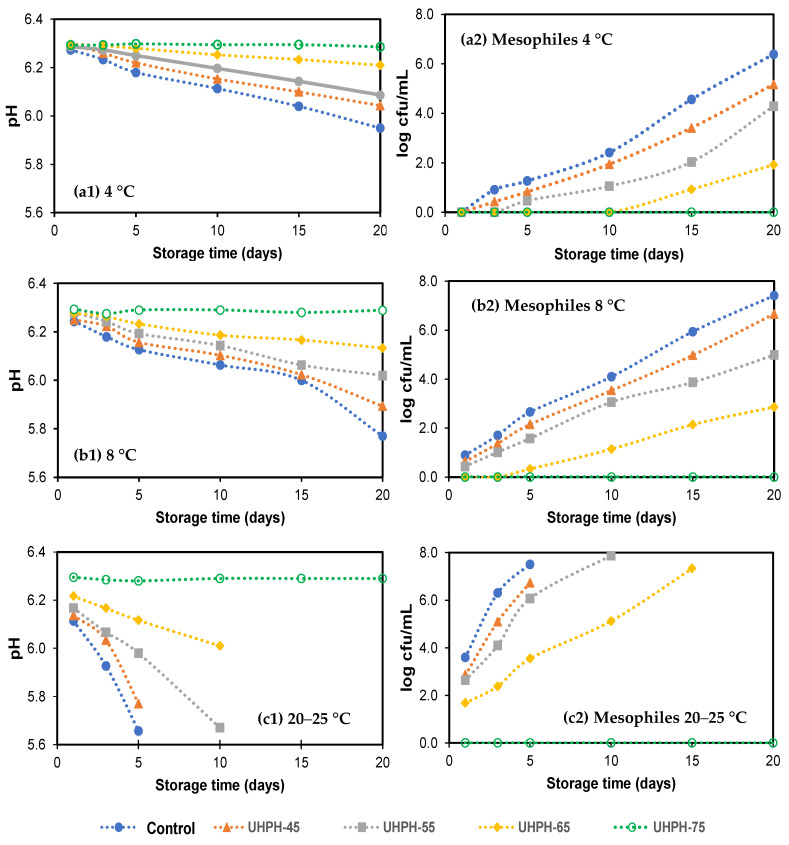
pH and mesophilic bacteria counts (cfu/mL) in untreated (control) and ultra-high-pressure-homogenized fish broths at 300 MPa at different inlet temperatures (45, 55, 65, and 75 °C) during storage at (**a1**,**a2**) 4 °C, (**b1**,**b2**) 8 °C, and (**c1**,**c2**) room temperature (20–25 °C).

Figure 1 shows the evolution of mesophilic aerobic bacteria counts (cfu/mL) and pH during 20 days of storage at 4 and 8 °C and at room temperature (20–25 °C). After the cooking process and UHPH treatments, counts of all the microbial groups analyzed were below the quantification limit of 1 cfu/mL. However, only *Enterobacteriaceae* counts remained below the quantification limit in all treatments applied (data not shown). As expected, counts of mesophilic aerobic microorganisms in the broths kept under refrigeration conditions were higher in the untreated fish broth (control). After 15 and 10 days at 4 and 8 °C, respectively, counts remained below 5 log cfu/mL considered the rejection limit value in foods prepared with heat treatment in the Spanish Regulation [31]. In UHPH-45 fish broths stored at 4 °C, bacteria counts were equal to or greater than 5 log cfu/mL on day 20. In contrast, in the UHPH-55 and UHPH-65 fish broths, counts remained below 5 log cfu/mL, reaching a maximum value of approximately 2 log cfu/mL in the UHPH-65 fish broths at the end of storage (Figure 1). In fish broths stored at 8 °C, a similar trend was obtained. However, the growth rate of mesophilic aerobic microorganisms was higher, reaching counts of 5 log cfu/mL on day 15 in the UHPH-45 fish broths and day 20 in the UHPH-55 fish broths. At the end of storage time, in the UHPH-65 fish broths, counts were around 3 log cfu/mL (Figure 1). During storage at 20–25 °C, a rapid increase in the counts was observed, so the UHPH-45 and UHPH-55 fish broths reached the rejection limit in less than 3 days, while the UHPH-65 fish broths lasted for up to 10 days. The growth of microorganisms was reflected in a gradual decrease in pH, such that a pH of 5.9–6 would be related to the microbial rejection limit of 5 log cfu/mL.

The spore count at both temperatures was minimal, with maximum counts of 1.5 to 2.5 log spores/mL at 4 and 8 °C in the control or UHPH-45 and UHPH-55 fish broths, coinciding with the beginning of the exponential phase of the count of mesophilic aerobic microorganisms (Figure S3). Microorganisms come from raw materials and environmental contamination during and after broth production [32]. The cooking temperatures used in the study (boiling ~100 °C, ~30 min) explain the persistence of sporulated microorganisms, which will grow later during refrigeration storage and at room temperature in untreated fish broth. UHPH treatments at inlet temperatures up to 65 °C were ineffective in eliminating sporulated microbiota of fish broths; however, a notable reduction in the microbiota was achieved, prolonging the shelf-life of the fish broth stored at refrigerated temperatures. Similar results were obtained in other liquid matrices, such as cow milk, soy or tiger nut beverages, or apple juice [13,14,18,33].

A stable microbiological product was obtained in UHPH-75 fish broths. Counts were below the quantification limit (<1 cfu/mL) at all storage temperatures. In addition, no growth was observed after the sterility test incubation at 30 and 55 °C for 21 days. Other authors have obtained total inactivation by UHPH technology in other liquid food matrices. Poliseli-Scopel et al. (2012) [10] working with soy beverage and Amador-Espejo et al. (2014) [34] with cow’s milk treated at 200 and 300 MPa with inlet temperatures of 55, 65, 75, and 85 °C obtained sterile products by applying 300 MPa at inlet temperatures equal to or greater than 75 °C. However, Valencia-Flores et al. (2013) [11] obtained sterile almond beverages at 300 MPa at an inlet temperature of 65 °C. In all these studies, the effect of cavitation phenomena, shear stress, shear, changes in kinetic energy, etc., were highlighted as a factor of microbial lethality when passing through the high-pressure valve, as well as the increase in temperature because of adiabatic heating for <0.5 s. In the case of soy and almond beverages, the increase in temperature after the valve (T_2_) was ~135 °C in the treatment at 300 MPa at T_i_ of 75 °C [17]. However, in this survey, the temperature reached in UHPH-75 fish broths was T_2_~128 °C. The temperature after the valve and the associated physical effects varies from one piece of equipment to another, depending on the design of the valves. Moreover, the microbial lethality obtained using this technology is also affected by the product’s fluidity-viscosity and the microbiota of processed food [17].

### 3.3. Effect of UHPH on the Particle Size

**Figure 2:**
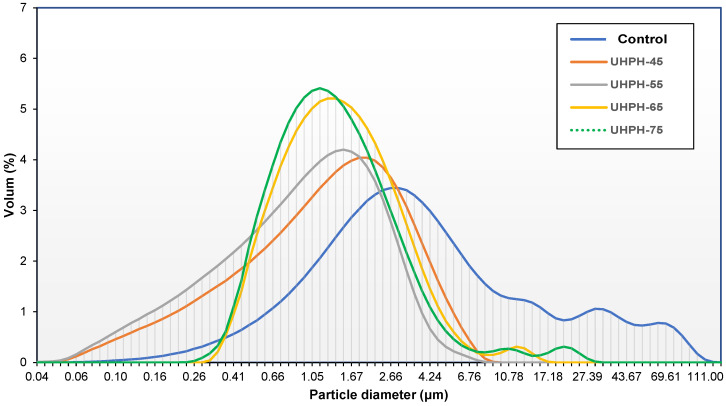
Particle size distribution curves of untreated (control) and ultra-high-pressure-homogenized fish broths at 300 MPa at different inlet temperatures (45, 55, 65, and 75 °C).

Untreated fish broth (control) showed a polydisperse distribution, with the main peak between 0 and 10 µm in diameter (corresponding to 77.1% of particles), followed by a set of minor peaks with a diameter between 11 and 120 µm. The parameters D_50_ and D_90_ were ~3.5 and ~14 µm, respectively, while the fish broths treated with UHPH have diameters lower than 1.45 µm and 3.70 µm, respectively (Table 2). Similar results were found in untreated tiger nut, soy, and almond vegetable drinks [9,10,11,33]. In UHPH-treated fish broths, different distribution curves were observed due to the reduction in particle size, with 98% of the particles having a diameter of <10 µm (Figure 2). The application of UHPH treatments reduced significantly (*p* < 0.05) the size of the particles, and values of parameters D_50_, D_90_, d_3,2_, and d_4,3_ decreased significantly (*p* < 0.05); see Table 2. However, specific differences in the distribution curves were noted as a function of inlet temperature. Specifically, a monomodal distribution curve was obtained in the UHPH-45 and UHPH-55 fish broths. In comparison, the UHPH-65 and UHPH-75 fish broths showed a monomodal curve distribution with a tail of minor peaks corresponding to particles of greater diameter, probably due to the formation of aggregates of particles (Figure 2). This phenomenon was appreciated in parameters d_3,2_ and d_4,3_ (Table 2). The increase in inlet temperature could probably cause a higher denaturation of the protein, a conformational change, and, consequently, a decrease in their solubility. Moreover, new interactions between the protein and the other particles in the suspension were established, with the consequent formation of particle aggregates [9].

### 3.4. Effect of UHPH on the Color and Sensorial Characteristics

The luminosity (L*) and b* values of untreated fish broth were lower (less white) and higher (more yellow), respectively, than of the UHPH-65 and UHPH-75 fish broths, while in the a* value (higher red tendency), differences were observed concerning all UHPH treatments (Table 3). In UHPH-treated fish broths, luminosity increased, and the b* value gradually decreased with increasing inlet temperature (*p* < 0.05), although no differences were observed in the a* value. The increase in luminosity (L*) of the UHPH-treated fish broths as the inlet temperature increases could be associated with the increase in light diffraction caused by the greater number of little particles in suspension [18]. The effect of UHPH on color, especially luminosity, depends significantly on the matrix and the UHPH treatment. Although in tiger nut and soy beverages and milk with variable treatments at 200–300 MPa at inlet temperatures between 20 and 85 °C [9,10,14,34], similar results were observed. However, in another study carried out with soymilk, a decrease in luminosity was obtained [33]. The differences between untreated and UHPH-treated fish broths in luminosity and the b* value were reflected in the parameter ∆E* (Table 3). In the UHPH-treated fish broth, those treated at higher inlet temperatures were the most different, the order of these differences being T_i_ 45 °C = 55 °C ≤ 65 °C < 75°C. In this study, the ΔE* value was 3.45 in the fish broths treated at UHPH-75, considered a value that would already be appreciable by the human eye (ΔE* > 3; Figure S4) [35].

A triangular similarity test was applied for sensorial analysis to evaluate perceptible sensory differences between UHPH-75 fish broths, selected because sterility was achieved, and untreated fish broth. Of the 18 judges selected, only 3 correctly identified the difference. After statistical analysis, the conclusion would be that less than 90% of consumers would be able to detect differences between untreated and UHPH-treated fish broths.

### 3.5. Composition and Antioxidant Properties of Untreated and UHPH-75 Fish Broths

The different broths were physiochemically characterized to evaluate their nutritional profile and the antioxidant activity of their components before UHPH treatments. Additionally, analyses were also performed on the UHPH-75-treated fish broths.

The pH value was similar in all fish broth formulations, with an approximate value of 6.20. Few bibliographical references are related to the composition of fish broths, possibly due to the high variability in their production process. A possible comparison could be obtained from the United States Department of Agriculture (USDA) database of nutrients. The fish broth composition of this database is 4% dry matter, 2% protein, 0.6% lipid, and 0.9% minerals [36]. The fish broth formulation with monkfish heads and hake pieces was the most similar in protein and lipid content. Dry matter content was higher in fish broths with hake pieces incorporated, and a significant increase (*p* < 0.05) in total lipid content was observed when rock fish was added (Table 4). Fish species are usually grouped into fat, semi-fat, and low-fat (lean), based on the percentage of muscle fat per 100 g of wet weight. In our case, both monkfish (*Lophius piscatorius*) and hake (*Merluccius merluccius)* are species considered lean or white fish, with a muscle lipid content of less than 1% [37,38]. The contribution of lipids in broths made with rock fish would be mainly associated with red mullet (*Mullus* spp.), typically consumed in the Spanish territory, with a lipid content between 5.32 and 8.32% [37,38].

Three parameters related to antioxidant activity, total polyphenols, ferric-reducing antioxidant capacity (FRAP), and Trolox-equivalent antioxidant capacity (TEAC), were evaluated. The three main components (fish, vegetables, and seasoning spices) used in the fish broth formulations have components with antioxidant activity. As seen in Table 4, the results obtained in the total polyphenol content did not show a correlation with the other parameters of antioxidant activity, as other authors have already observed in heat-treated fresh vegetables [39]. Consequently, other substances may play an important role in antioxidant activity [40]. When rock fish was included in the formulation, the total content of phenols and TEAC was generally significantly higher, especially in the formulations with 750 and 600 g of fish, decreasing in untreated and UHPH-75-treated fish broths with 450 g of fish and those elaborated exclusively with monkfish heads. Moreover, no statistical differences were observed in the nutritional composition and antioxidant activity between untreated and UHPH-75 fish broths (Table 4). Preserved antioxidant capacity has also been observed in apple or orange juice [13,28], although in these studies, the inlet temperature was either 8 or 20 °C.

In marine species, antioxidant substances are fat-soluble or water-soluble metabolites of low molecular mass, such as ascorbic acid, tocopherols, carotenoids, coenzyme Q, glutathione (GSH), thiols, bilirubin, and uric acid [41,42], the liposoluble antioxidant substances being higher in red muscle than in white muscle [43]. In addition, the components of rock fish, such as triglides, scorpenids, and mulids, contain carotenoid substances, such as astaxanthin, which could contribute to an increase in antioxidant content [44,45].

The vegetables and spices used in fish broth also have components with strong antioxidant activity, such as ascorbic acid, tocopherols, carotene, and/or phenolic compounds [39,46,47], being present in onion, parsley, carrot, celery, leek [48], oregano, and black peppercorns [49]. However, their activity can be affected by the thermal treatments used in elaborating the broths. Thus, studies on different fresh vegetables have shown that due to heat treatments, the antioxidant capacity could be reduced [50] or not altered or increased [47]. Among the compounds with antioxidant activity, flavonoids present higher stability to thermal treatments [39,46]. In this group, flavonols, such as quercetin, are present in onions, and flavones, such as apigenin and luteolin, are present in parsley, celery, and oregano [51]. Preparing vegetables before cooking also affects the content of antioxidant substances due to the structural modifications caused in their processing [50], such as the cell disruption caused by the cut, affecting the amount and composition of polyphenols [46]. The amount of antioxidant compounds leached during boiling varies depending on the characteristics of the products [48], so fish broth, depending on the raw material used, could retain a high content of phytochemicals.

Fish broths had low antioxidant activity and reduced content of phenolic compounds when compared to fresh products due to different factors, such as the reduced quantity of ingredients with the highest concentration of antioxidant substances (vegetables and spices), the effect of thermal treatment, and the possibility of interactions between complex components [24], especially in the fish broth with hake pieces, which can cause a variation in the antioxidant response. However, compared to other broths, mainly of vegetable origin [48], the values obtained are similar or even higher, possibly due to the contribution of fish to these parameters.

## 4. Conclusions

Applying a systematic methodology based on sensory analysis and nutritional composition has been a strategy adequate to reduce the variability related to the ingredients of fish broth for evaluating the effect of UHPH technology, allowing standardization of the elaboration process and selection of fish broth with the best organoleptic and nutritional characteristics at the lowest cost.

UHPH treatments at 300 MPa at inlet temperatures between 45 and 75 °C increased the shelf-life of fish broths compared to untreated fish broth. The microbiological shelf-life of untreated fish broth during cold storage at 4 and 8 °C improved from 10–15 days to more days at 20 °C after UHPH processing at 300 MPa. Moreover, when UHPH treatments at an inlet temperature of 75 °C were applied, the sterility of the product was achieved, remaining stable, even when the fish broth was stored at room temperature. Physical stability also improved with UHPH treatments, and color was the attribute more affected, appreciating instrumentally and visually higher luminosity and lower yellowness in the fish broth. For its higher microbiological stability, the treatment of choice would be UHPH treatment at 300 MPa at an inlet temperature of 75 °C. In this broth, regarding the untreated broth, no differences were observed in sensory perception, and its properties of nutritional composition and antioxidant activity were maintained. In conclusion, UHPH processing could be a promising technology for obtaining fish broth with an improved microbiological shelf-life, preserving its nutritional, antioxidant, and sensory properties.

## Figures and Tables

**Table 1:** Fish broth formulations: ingredients and quantity.

Ingredients	Quantity (g/L) of Ingredients												
	1	2	3	4	5	6	7	8	9	10	11	12	13
Salt	10	5.3	5.3	5.3	5.3	5.3	5.3	5.3	5.3	5.3	5.3	5.3	5.3
Sunflower oil	24	12	12	12	12	12	12	12	12	12	12	12	12
Monkfish heads	750	750	750	750	750	750	750	750	375	375	250	300	225
Rock fish	0	0	0	0	0	0	0	0	0	375	250	300	225
Hake	0	0	0	0	0	0	0	0	375	0	250	0	0
Onion	30	40	55	80.3	30	30	30	55	55	55	55	55	55
Carrot	25	49	60	64.5	25	25	25	60	60	60	60	60	60
Leek	15	13	15.8	15.5	15	15	15	15.8	15.8	15.8	15.8	15.8	15.8
Lemon	10	8	9	8.1	10	10	10	9	9	9	9	9	9
Celery	7	7	10	7	7	7	7	10	10	10	10	10	10
Parsley	5	5	6.2	5	5	5	5	6.2	6.2	6.2	6.2	6.2	6.2
Oregano	0.3	0.3	0.3	0.3	0.4	0.5	0.6	0.5	0.5	0.5	0.5	0.5	0.5
Peppercorns	2	2	2	2	0.9	1.3	1.4	1.3	1.3	1.3	1.3	1.3	1.3

**Table 2:** Particle size parameters (d_4,3_, d_3,2_, D_50_, and D_90_) of untreated (control) and ultra-high-pressure-homogenized fish broths at 300 MPa at different inlet temperatures (45, 55, 65, and 75 °C).

Treatments	d_4,3_	d_3,2_	D_50_	D_90_
Control	5.85 ± 0.17 ^d^	1.17 ± 0.20 ^b^	3.67 ± 0.11 ^b^	14.85 ± 1.53 ^b^
UHPH-45	1.89 ± 0.13 ^b^	0.65 ± 0.08 ^a^	1.35 ± 0.24 ^a^	3.70 ^a^ ± 0.82 ^a^
UHPH-55	1.62 ± 0.07 ^a^	0.53 ± 0.06 ^a^	1.04 ± 0.37 ^a^	3.10 ± 0.62 ^a^
UHPH-65	1.96 ± 0.04 ^bc^	1.23 ± 0.01 ^b^	1.45 ± 0.12 ^a^	3.59 ± 0.51 ^a^
UHPH-75	2.10 ± 0.08 ^c^	1.17 ± 0.03 ^b^	1.35 ± 0.14 ^a^	3.49 ± 0.20 ^a^

Data are presented as the mean value of three replications ± standard deviation. ^a–c^ Different superscript letters in the same column indicate significant differences (*p* < 0.05) between samples.

**Table 3:** Color parameters (L*, a*, b*, and ∆E*) in untreated (control) and ultra-high-pressure-homogenized fish broths at 300 MPa at different inlet temperatures (45, 55, 65, and 75 °C).

Treatments	*L*	a*	b*	∆E* ^1^
Control	48.43 ± 0.27 ^a^	1.28 ± 0.23 ^b^	15.25 ± 0.23 ^d^	-
UHPH-45	48.86 ± 0.57 ^a^	−0.40 ± 0.03 ^a^	14.89 ± 0.11 ^cd^	1.76 ± 0.17 ^a^
UHPH-55	49.44 ± 0.54 ^ab^	−0.23 ± 0.05 ^a^	14.47 ± 0.10 ^bc^	1.93 ± 0.28 ^ba^
UHPH-65	50.20 ± 0.56 ^bc^	−0.24 ± 0.03 ^a^	14.27 ± 0.12 ^b^	2.48 ± 0.39 ^b^
UHPH-75	51.09 ± 0.15 ^c^	−0.16 ^a^ ± 0.05 ^a^	13.43 ± 0.12 ^a^	3.45 0.11 ^c^

Data are presented as the mean value of three replications ± standard deviation. ^1^ The total color differences were calculated by taking reference to the control fish broth. ^a–d^ Different superscript letters in the same column indicate significant differences (*p* < 0.05) between samples.

**Table 4:** Chemical composition, pH, antioxidant capacity (FRAP and TEAC), and phenolic components of the different fish broth formulations and fish broth ultra-high-pressure-homogenized at 300 MPa at an inlet temperature of 75 °C.

Fish Broth ^1^	pH	Protein (g/100 mL)	Lípids (g/100 mL)	Ash (g/100 mL)	Dry Matter (g/100 mL)	FRAP ^2^ (mM)	TEAC ^3^ (mM)	Phenolic Compounds (mg EAG/L) ^4^
MH (750 g)	6.20 ± 0.02 ^a^	1.08 ± 0.06 ^ab^	0.86 ± 0.09 ^a^	0.92 ± 0.23 ^a^	3.19 ± 0.59 ^b^	1.33 ± 0.06 ^ab^	2.52 ± 0.18 ^bc^	630.06 ± 16.48 ^b^
MH-HP (750 g)	6.26 ± 0.01 ^c^	2.14 ± 0.52 ^de^	1.06 ± 0.10 ^a^	1.71 ± 0.21 ^d^	5.61 ± 0.14 ^f^	1.09 ± 0.03 ^a^	1.96 ± 0.10 ^a^	384.34 ± 32.15 ^a^
MH-HP-RF (750 g)	6.25 ± 0.01 ^bc^	1.78 ± 0.52 ^d^	1.63 ± 0.84 ^b^	1.45 ± 0.11 ^c^	5.34 ± 0.10 ^e^	1.30 ± 0.23 ^ab^	2.20 ± 0.07 ^ab^	589.52 ± 24.23 ^b^
MH-RF (750 g)	6.22 ± 0.00 ^ab^	1.57 ± 0.36 ^c^	2.19 ± 0.83 ^d^	1.31 ± 0.04 ^bc^	5.12 ± 0.45 ^e^	1.62 ± 0.1 ^b^	2.84 ± 0.11 ^c^	817.93 ± 09.31 ^d^
MH-RF (600 g)	6.25 ± 0.01 ^bc^	1.29 ± 0.26 ^b^	1.97 ± 0.44 ^cd^	1.26 ± 0.12 ^bc^	4.75 ± 0.56 ^d^	1.34 ± 0.25 ^ab^	2.83 ± 0.16 ^c^	755.52 ± 10.38 ^c^
MM-RF (450 g)	6.23 ± 0.02 ^bc^	1.02 ± 0.16 b^b^	1.11± 0.23 ^a^	1.05 ± 0.06 ^a^	2.89 a ± 0.44 ^ab^	1.28 ± 0.09 ^ab^	2.75 ± 0.26 ^c^	635.35 ± 21.35 ^b^
UHPH-75 fish broths	6.13 ± 0.12 ^abc^	0.97 ± 0.18 ^ab^	1.04 ± 0.24 ^a^	0.93 ± 0.04 ^a^	2.75 ± 0.38 ^ab^	0.83 ±0.35 ^a^	3.06 ±0.22 ^c^	602.50 5 ± 97.3 ^b^

Data are presented as the mean value of three replications ± standard deviation. ^1^ Fish broth formulations: MH: monkfish heads; HP: hake pieces; RF: rock fish. The total quantity of fish added (g/L). ^2^ FRAP: ferric-reducing antioxidant capacity; ^3^ TEAC: Trolox-equivalent antioxidant capacity; ^4^ EAG: equivalent of gallic acid. ^a–f^ Different superscript letters in the same column indicate significant differences (*p* < 0.05) between samples.

## Data Availability

Data is contained within the article and supplementary material.

## Supplementary Materials

The following supporting information can be downloaded at https://www.mdpi.com/article/10.3390/foods11243969/s1: Figure S1: Experimental flow chart for selecting the best fish broth formulation. FRAP: Ferric Ion Reducing Antioxidant Capacity; TEAC: Trolox Equivalent Antioxidant Capacity; Figure S2: Experimental flow chart for evaluating the effect of the application of ultra-high pressure homogenized (UHPH) treatments at 300 MPa at inlet temperatures (T_i_) between 45 and 75 °C on microbiological, physical and sensorial characteristics of fish broth. FRAP: Ferric Ion Reducing Antioxidant Capacity. TEAC: Trolox Equivalent Antioxidant Capacity; Figure S3: Mesophilic sporulated bacteria counts (cfu/mL) in untreated (control) and ultra-high pressure homogenized fish broth at 300 MPa at different inlet temperatures (45, 55, 65 and 75 °C) during storage at (a) 4 °C, (b) 8 °C and (c) room temperature (20–25 °C); Figure S4: Image of the differences in color between the untreated fish broth (control) and treated by ultra-high pressure homogenization (UHPH) at 300 MPa at inlet temperature 75 °C. Table S1: Olfactory-gustatory profile of the base formulation of the fish broth (Broth 1). Evaluation of each compound intensity in the taste, aftertaste and persistence (scale 0–5) and overall impression (scale 0–3) of the fish broth (UNE 87017:1992); Table S2: Sorting test of fish broth formulations based on overall impression (scale 0–3) depending on the content of vegetables or species (UNE-ISO 8587:2010); Table S3: Sorting test of fish broth formulations based on the acceptability of the intensity of taste, smell and overall impression (scale 0–3) depending on the kind of fish (UNE-ISO 8587:2010); Table S4: Sorting test of fish broth formulations based on the acceptability of the intensity of taste. smell and overall impression (scale 0–3) depending on the quantity of monkfish-rock fish) (UNE-ISO 8587:2010).
