# Supplementary material for: Effect of Ultra-High-Pressure Homogenization Processing on the Microbiological, Physicochemical, and Sensory Characteristics of Fish Broth
Source: Foods. 2022 Dec 8;11(24):3969. doi: 10.3390/foods11243969 (PMC9777534; doi:10.3390/foods11243969)
Supplement: Supplementary file 1 [file foods-11-03969-s001.zip › foods-2041037-supplementary.pdf]

SUPPLEMENTARY MATERIAL

Article

Effect of Ultra-High-Pressure Homogenization Processing on the Microbiological, Physicochemical, and Sensory Characteristics of Fish Broth

Sonia Genuina Moisés [1], Buenaventura Guamis [1], Artur Xavier Roig-Sagués [1], Idoia Codina-Torrella [2] and Maria Manuela Hernández-Herrero [1,*]

[1] Departament de Ciència Animal i dels Aliments, Facultat de Veterinària, Centre d'Innovació, Recerca i Transfèrencia en Tecnologia dels Aliments (CIRTTA), XaRTA, TECNIO-CERTA, MALTA-Consolider Team, Universitat Autònoma de Barcelona, 08193 Bellaterra, Spain
[2] Departament d'Enginyeria Agroalimentària i Biotecnologia, Edifici D4C, Esteve Terradas, 8, 08860 Castelldefels, Spain
* Correspondence: manuela.hernandez@uab.cat

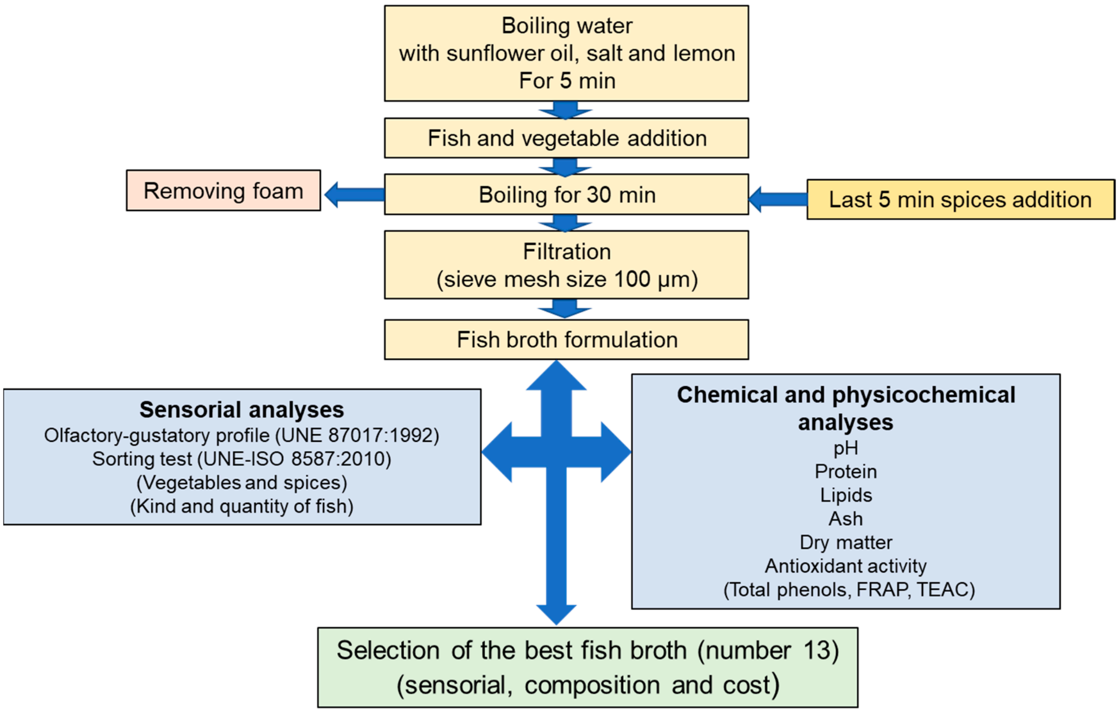

Figure S1. Experimental flow chart for selecting the best fish broth formulation. FRAP: Ferric Ion Reducing Antioxidant Capacity; TEAC: Trolox Equivalent Antioxidant Capacity.

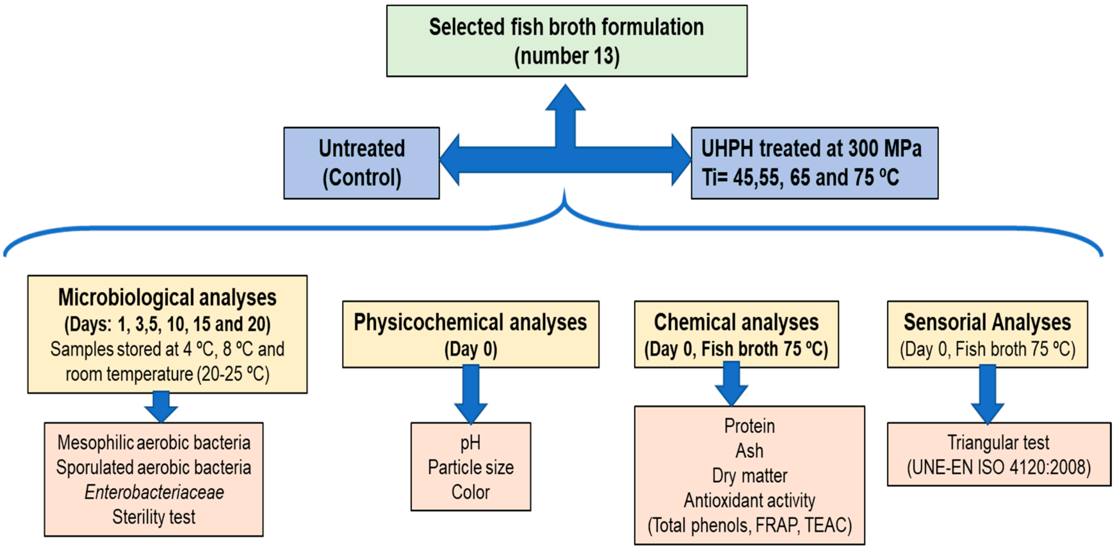

Figure S2. Experimental flow chart for evaluating the effect of the application of ultra-high pressure homogenized (UHPH) treatments at 300 MPa at inlet temperatures (Ti) between 45 and 75 °C on microbiological, physical and sensorial characteristics of fish broth. FRAP: Ferric Ion Reducing Antioxidant Capacity. TEAC: Trolox Equivalent Antioxidant Capacity.

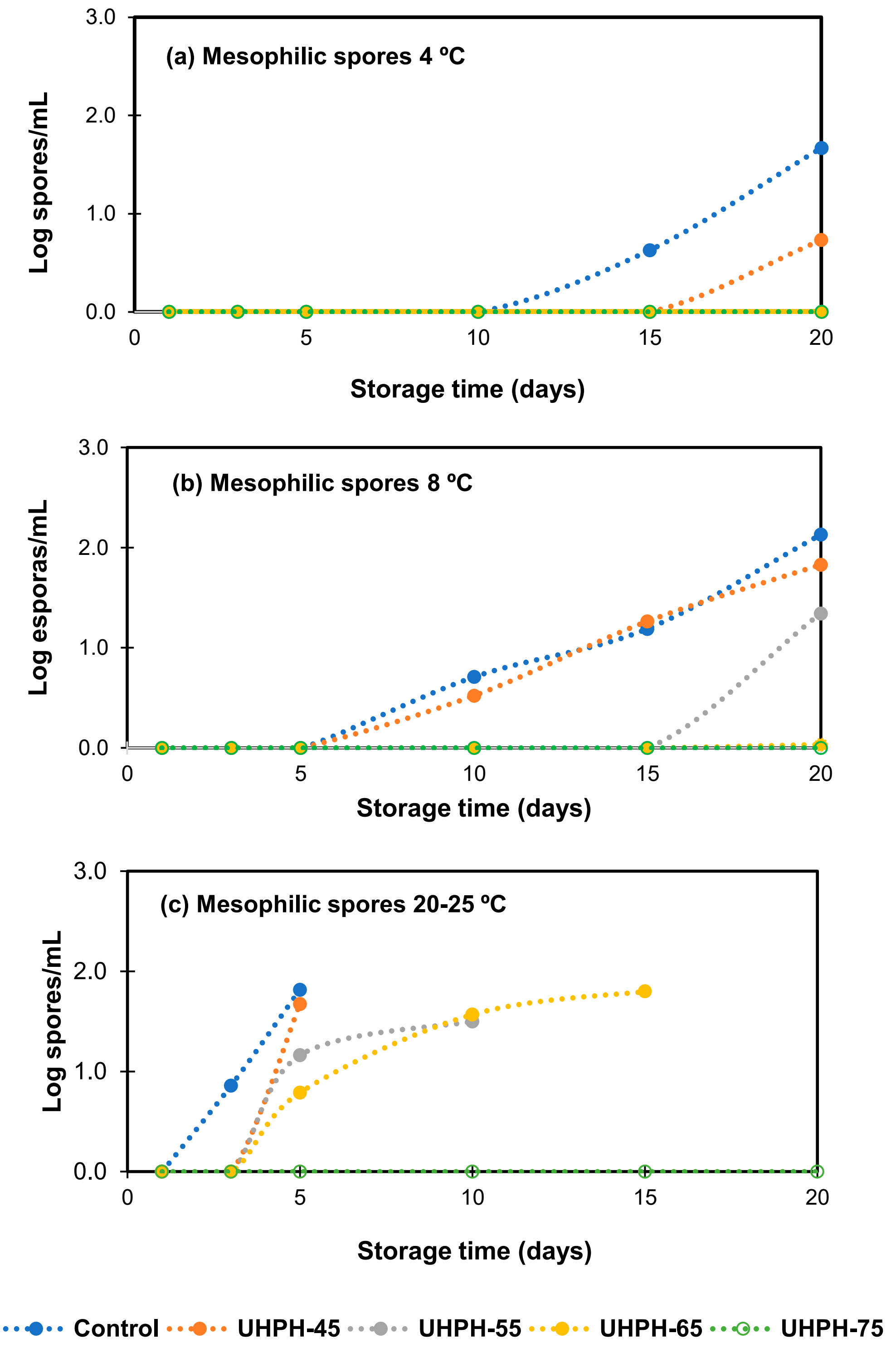

Figure S3. Mesophilic sporulated bacteria counts (cfu/mL) in untreated (control) and ultra-high pressure homogenized fish broth at 300 MPa at different inlet temperatures (45, 55, 65 and 75 °C) during storage at (a) 4 °C, (b) 8 °C and (c) room temperature (20-25 °C).

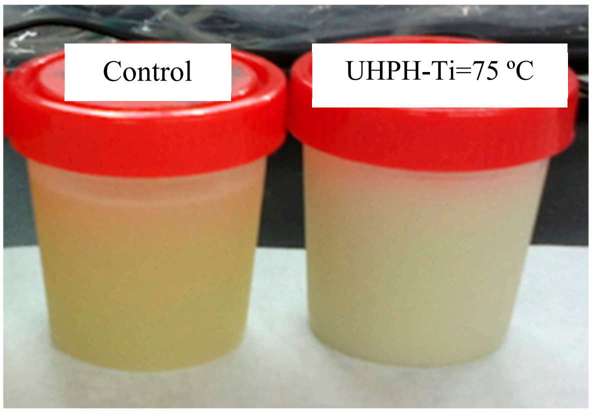

Figure S4. Image of the differences in color between the untreated fish broth (control) and treated by ultra-high pressure homogenization (UHPH) at 300 MPa at inlet temperature 75 ºC.

Table S1. Olfactory-gustatory profile of the base formulation of the fish broth (Broth 1). Evaluation of each compound intensity in the taste, aftertaste and persistence (scale 0-5) and overall impression (scale 0-3) of the fish broth (UNE 87017:1992).

Attribute	Score
**Taste**	
Salt	5
Sunflower oil	5
Monkfish	4
Onion	2
Carrot	2
Leek	3
Lemon	3
Celery	2
Parsley	2
Oregano	2
Pepper	5
**Aftertaste**	2
**Persistence**	2
**Overall impression**	2

Table S2. Sorting test of fish broth formulations based on overall impression (scale 0-3) depending on the content of vegetables or species (UNE-ISO 8587:2010).

Broth	Quantity of ingredients (g/L)	Sum (R_i)[1]
	**Vegetables**	
1	92	19[a]
2	122	30[ab]
3	180	31[ab]
4	156	40[b]
	**Spices**	
1	2.3	18[a]
5	1.3	34[bc]
6	1.8	38[c]
7	2.0	30[ab]

[1]R_i: The score gives the sum of 18 panelists.

[a-c]: Different letters in the same column are significantly different ($p < 0.05$)

Table S3. Sorting test of fish broth formulations based on the acceptability of the intensity of taste, smell and overall impression (scale 0-3) depending on the kind of fish (UNE-ISO 8587:2010).

Broth[1]	Sum (R_i)[2]		
	**Smell**	**Taste**	**Overall impression**
**(8) MH**	30.0^{ab}	25.0^{ab}	19.5^{a}
**(9) MH-HP**	31.0^{ab}	34.0^{bc}	42.5^{b}
**(10) MH-RF**	54.0^{c}	54.5^{d}	54.0^{b}
**(11) MH-HP-RF**	41.5^{bc}	45.0^{cd}	43.0^{b}

[1]Fish broth formulations: MH: monkfish heads; HP: hake pieces; RF: Rock fish.

[2]R_i: The score gives the sum of 18 panelists.

$^{a-d}$: Different letters in the same column are significantly different ($p < 0.05$)

Table S4. Sorting test of fish broth formulations based on the acceptability of the intensity of taste. smell and overall impression (scale 0-3) depending on the quantity of monkfish-rock fish) (UNE-ISO 8587:2010).

Broth	Sum $(R_i)^2$		
	Smell	Taste	Overall impression
(10) 750 g	26.5[a]	27.0[a]	25.0[a]
(12) 600 g	30.0 [a]	33.5[a]	30.0[a]
(13) 450 g	33.5[a]	29.5[a]	35.0[a]

[1]R_i: The score gives the sum of 18 panelists.

[a] : Different letters in the same column are significantly different ($p < 0.05$)
